# Mercury exposure in the Norwegian Mother, Father, and Child Cohort Study – measured and predicted blood concentrations and associations with birth weight

**DOI:** 10.1016/j.heliyon.2024.e30246

**Published:** 2024-04-25

**Authors:** Kristine Vejrup, Anne Lise Brantsæter, Ida H. Caspersen, Line S. Haug, Gro D. Villanger, Heidi Aase, Helle K. Knutsen

**Affiliations:** a Institute of Military Epidemiology, Norwegian Armed Forces Joint Medical Serviced, Norway; b Department of Food Safety and Centre for Sustainable Diets, Norwegian Institute of Public Health, Norway; c Centre for Fertility and Health, Norwegian Institute of Public Health, Norway; d Department of Child Health and Development, Norwegian Institute of Public Health, Norway

**Keywords:** Mercury, Fish intake, Prediction model, Birth weight, MoBa, Negative confounding

## Abstract

**Background:**

Blood total mercury concentration (BTHg) predominantly contains methyl Hg from seafood, and less inorganic Hg. Measured BTHg is often available only in a small proportion of large cohort study samples. Associations between estimated dietary intake of total Hg (THg) and lower birth weight within strata of maternal seafood intake was previously reported in the Norwegian Mother, Father, and Child Cohort Study (MoBa). However, maternal seafood consumption was associated with increased birth weight, indicating negative confounding by seafood in the association between THg intake and birth weight. Using predicted BTHg as a proxy for measured BTHg, we hypothesized that predicted BTHg would be associated with decreased birth weight.

**Objectives:**

To develop and validate a prediction model for BTHg in MoBa and to examine the association between predicted BTHg and birth weight in the MoBa population.

**Methods:**

Using linear regression, measured maternal BTHg (n = 1437) was used to build the best fitting model (highest R-squared value). Model validation (n = 1436) was based on correlation and weighted Kappa (Кw). Associations between predicted BTHg in the MoBa population (n = 86,775) or measured BTHg (n = 3590) and birth weight were assessed by multivariate linear regression models.

**Results:**

The best fitting model had R-squared = 0.3 and showed strong correlation (r = 0.53, p < 0.001) between predicted and measured BTHg. Cross-classification (quintiles) showed 73 % correctly classified and 3.3 % grossly misclassified, with Кw of 0.37. Measured BTHg was not associated with birth weight. Predicted BTHg was significantly associated with higher birth weight. There were no trends in birth weight with increasing quintiles of measured or predicted BTHg after stratification into high or low seafood consumption.

**Conclusions:**

The results indicate that prediction of BTHg did not overcome negative confounding of the association between Hg exposure and birth weight by seafood intake. Furthermore, effect on birth weight of toxicological concern is unexpected in our observed BTHg range.

## Highlights

• Model predicted blood total Hg was highly correlated with measured blood total Hg.
• Measured blood total Hg (n = 3590) was not associated with birth weight.
• Predicted blood total Hg (n = 86,775) was associated with higher birth weight.

## Graphical abstract

**Figure:**
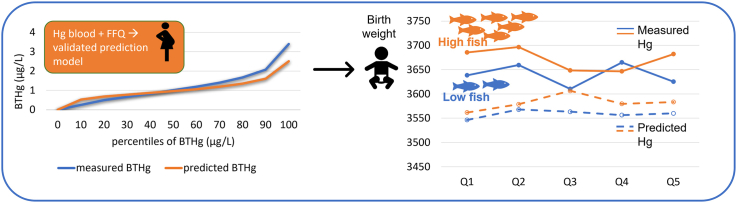

## Abbreviations

BTHg: Blood total mercury
IHg: Inorganic mercury
MeHg: Methyl mercury
MoBa: The Norwegian Mother, Father, and Child Cohort Study
TWI: Tolerable weekly intake

## 1 Introduction

Exposure to methyl mercury (MeHg) during pregnancy may cause impairment of cognitive development or increase risk of neurodevelopmental disorders in the child (1,2]. Furthermore, there are indications that MeHg exposure restricts prenatal growth and lowers birth weight [[3], [4], [5], [6]]. Toxicokinetic modelling and assessment factors have been used to derive the safe dietary exposure level at which no appreciable health effects are expected to occur in any part of the population [1,2,7]. Both previous and more recent dietary exposure assessments from Europe indicate that MeHg intake is generally below 1.3 μg/kg body weight per week, which is the tolerable weekly intake (TWI) established by EFSA in 2012 [2,[8], [9], [10]]. However, parts of the population with high consumption of certain fish species have exposure above the tolerable intakes [[11], [12], [13]]. Still, it is debated whether MeHg exposure from maternal fish consumption below the TWI of 1.3 μg/kg body weight per week might affect neurodevelopment in children, considering the possibility that these effects remain undetected in birth cohorts because of the beneficial effects of fish consumption per se.

Dietary assessment in cohort studies can cover a large number of participants and have the potential to encompass a wide range of exposures to MeHg and other contaminants. However, dietary intake estimates are associated with numerous uncertainties related to both the dietary reports and the contaminant concentrations in the foods consumed. Moreover, the internal concentrations of a contaminant will vary with its toxicokinetic properties related to absorption, distribution, and elimination. Measured levels of contaminants with long half-lives in the body, such as MeHg, provide more accurate estimates of the internal exposure. However, the available amount of biological material and analytical costs are well-known limitations. The combination of dietary assessment and measured levels in the body allows for validation of dietary-based estimates. If the predictive ability of such models is satisfactory, exposure estimates derived from dietary surveys can be used in studies of substantially larger sample size to examine associations with health outcomes.

Mercury (Hg) in seafood is predominantly present as MeHg and seafood is virtually the only source of MeHg exposure in the general population. Seafood is also an important source of beneficial nutrients such as long chained n-3 polyunsaturated fatty acids and iodine. Recognizing the toxic effects of MeHg on different health outcomes, several studies point to the challenges of disentangling the positive effect of nutrients associated with fish consumption from the risk from MeHg exposure, an issue denoted negative confounding [[14], [15], [16], [17], [18]]. To adjust for negative confounding appropriately, good data on fish and other seafood consumption, and independent data on Hg exposure such as level in blood or hair are needed.

Many studies show correlation between fish consumption and total mercury in blood (BTHg) or hair [11,19]. Whereas hair contains only MeHg and reflects MeHg exposure during hair growth, blood contains both inorganic Hg (IHg) and MeHg. Still, the concentration of MeHg is the largest fraction of BTHg, constituting on average around 80 % [20]. BTHg is recognized as a good marker of MeHg exposure in a fish-eating population, reflecting exposure during the recent months before sampling. MeHg in blood is predominantly found in the red blood cells, while IHg is predominantly found in blood plasma. IHg comes from food, demethylation of MeHg as well as other sources. In addition, amalgam in tooth restorations is a major source to IHg in the body, as metallic Hg vaporise from amalgam tooth fillings and is quickly oxidized to IHg [21].

We have previously reported associations between estimated dietary intake of THg and lower birth weight within strata of seafood intake in the Norwegian Mother, Father, and Child Cohort Study (MoBa) (n = 62,941) [5]. However, seafood consumption was overall associated with increased birth weight, indicating negative confounding [5]. Measured BTHg with adjustment for fish consumption would have provided a better basis for assessing associations, since it can be adjusted for dietary sources together with other relevant covariates. Measured BTHg has more recently become available for a subset of the MoBa participants. Predicted BTHg based on food consumption data and other covariates can provide a proxy for measured BTHg, and we hypothesized that predicted BTHg would be associated with decreased birth weight. The aims of the present study were therefore to develop and validate a prediction model for BTHg in pregnant MoBa women and to examine the associations between measured and predicted BTHg and birth weight in the MoBa population.

## 2 Materials and methods

### 2.1 Study subjects and data collection

MoBa is a prospective population-based pregnancy cohort conducted by the Norwegian Institute of Public Health [22]. The recruitment period was from 1999 to 2008 and women pregnant in their first trimester were recruited from all over Norway. Mothers could participate with more than one pregnancy and the cohort includes approximately 114,500 children, 95,200 mothers and 75,200 fathers. Participants were asked to answer questionnaires (available in Norwegian only) at regular intervals during pregnancy and after birth. The women consented to participation in 41 % of the pregnancies.

The dataset is linked to selected variables from the Medical Birth Registry of Norway (MBRN) [23]. The three MoBa questionnaires used in the present study were completed in gestational weeks 15 (general questionnaire covering maternal health, lifestyle, educational attainment, and background factors), week 22 (food frequency questionnaire (FFQ)) and week 30 (general questionnaire including question about the number of teeth with amalgam fillings). These questionnaires are available in English translation at the NIPH webpage [24].

The present study is based on version 10 of the quality assured data files released in 2017 and restricted to participants recruited from 2002 to 2008 because the MoBa food frequency questionnaire (FFQ) was included in the data collection from March 2002. The study sample used for building and validating the prediction model included 3590 pregnancies with BTHg measurements, of which 2981 samples from the Norwegian Environmental Biobank. The Norwegian Environmental Biobank is a sub-study within MoBa established with the aim of biomonitoring nutrients and environmental contaminants in MoBa participants. The sub-study included 2999 pregnant women (of which 2981 with measured BTHg) with available genetic data who had donated blood and urine samples and had responded to the first six questionnaires in MoBa [25]. The remaining 609 samples in the present study are randomly selected controls from a case-control study on child ADHD and ASD diagnoses. These 609 controls were frequency-matched to the ADHD case group on birth year and sex of the child [17].

The study population used for correlating predicted BTHg and estimated dietary Hg intake in the full cohort included only pregnancies where mothers had responded to the baseline questionnaire and the FFQ and was registered in the medical birth registry with a singleton delivery. Furthermore, only pregnancies with reported energy intake based on the FFQ between 4.5 and 20 MJ and with birth weight >600 g were included. The final datafile contained n = 86,775 pregnancies (72,891 unique mothers), of which n = 3588 had analyzed BTHg.

The composition of sub-samples used in the present paper is illustrated in Supplementary Figs. S1 and S2.

### 2.2 Ethics approval

The establishment of MoBa and initial data collection was based on a license from the Norwegian Data Protection Agency and approval from The Regional Committees for Medical and Health Research Ethics. All MoBa participants provided written informed consent before enrolment into the study. The MoBa cohort is currently regulated by the Norwegian Health Registry Act. The present study was approved by The Regional Committees for Medical and Health Research Ethics (2015/1346).

### 2.3 Hg in blood samples

Maternal blood specimens were obtained at the time of the routine ultrasound examination in gestational week 18 (mean 18.5, SD 1.3), and shipped by overnight mail to the MoBa biobank at ambient temperature [26]. At the biorepository, whole blood was aliquoted into two polypropylene deep-well plates (930 μl in each, ABgene, Surrey, UK) and stored at − 80° [27] in the NIPH Biobank. Maternal whole blood total Hg in samples used for the Norwegian Environmental Biobank part I (MoBa Etox) (n = 2981) were analyzed at the Department of Occupational and Environmental Medicine at Lund University, Sweden [25] while the 609 controls from the ADHD and ASD study were analyzed at the ALS laboratory in Luleå, Sweden [17].

At Lund University, BTHg was determined as total mercury in acid-digested samples by cold vapor atomic fluorescence spectrophotometry [28]. The level of detection (LOD, 0.07 μg/L) was calculated as three times the standard deviation (SD) of the blank. All analyzed samples were prepared and measured in duplicate, and the mean value was used. In samples with BTHg < LOD (n = 15) the values reported by the laboratory (ranging down to 0.031 μg/L) were used for statistical analyses. At the ALS laboratory in Luleå BTHg was determined using inductively coupled plasma sector field mass spectrometry (ICP-SFMS). The LOQ was 0.2 μg/L and concentrations in all 609 samples were above this LOQ. Internal quality control samples and procedure blanks were analyzed along with each batch of samples to ensure high quality of the determinations throughout the project. The analytical accuracy was verified towards certified reference material; Seronorm Trace elements whole blood L-1 and L-2 (SERO AS, Billingstad, Norway). More detailed information on analytical procedure can be found in Ref. [25] (appendix A) and [17] (Table S1).

BTHg concentrations were adjusted for laboratory effects using the Ratio-G batch adjustment described in Ref. [29]. We estimated the laboratory-adjusted Hg concentration M* for each participant j with the following equation:

$$M{*}_{j}=M_{j}\;x\;\left(\text{meanQCl}/{\text{meanQCl}}_{k}\right)\text{,}$$

Where M_j_ is the measured Hg concentration, mean QCl is the geometric mean of Hg concentrations in reference samples across all (both) laboratories, and mean QClk is the geometric mean of Hg in reference samples from laboratory k (i.e., in the laboratory in which sample of participant j was measured).

### 2.4 Dietary information

The MoBa participants completed a semi-quantitative FFQ in gestational week 22 that was designed to capture their average diet since the start of pregnancy [30]. Frequencies were converted to food intakes and FoodCalc and the Norwegian food composition table were used to calculate food and nutrient intakes. A validation study showed that the MoBa FFQ is a valid tool for estimating energy, nutrients and food amounts, including the intakes of different types of fish and seafood, as well as long chain marine fatty acid supplements, n-3 fatty acids and energy [[31], [32], [33]].

The women's habitual food consumption (g/day) were aggregated into 33 food groups. Intakes of the food group “seafood” were described in five distinct sub-groups denoted lean fish, semi-oily fish, other oily fish, salmon and trout (farmed) and shellfish. Fish with less than 2 % fat (e.g., cod, saithe and haddock) was defined as “Lean fish”, fish with 2–8% fat (e.g., wolffish, halibut and flounder) was defined as “semi-oily fish”, and fish with more than 8 % fat (e.g., herring and mackerel), was defined as “other oily fish”, and this group also included fish liver and roe, but excludes the oily species salmon and trout. Consumption of salmon and trout were addressed by specific questions in the FFQ. Salmon and trout were considered to be farmed as the consumption of wild salmon and trout is very low. The seafood sub-group “shellfish” includes shrimp, crab, and bivalves. Total seafood intake was calculated as the sum of these five sub-groups reported as bread spread and dinner. The fish intake from composite fish products (e.g., fish cakes or fish au gratin) was calculated based on recipes. Concentrations of total Hg (THg) in Norwegian seafood and other food items has been compiled in a database [12]. Lower bound concentrations (results below levels of quantification were set to zero) were used for dietary exposure estimates. The THg contributed by the two food groups “eggs” and “bread and cereals” exceeded one percent of the total intake and were presented separately, as were also the seafood sub-groups. Food groups that contributed 1 % or less THg to diet were collected in the group “Other food products”. This group comprised meat and animal products, chocolate and sweets, fruit, and vegetables.

### 2.5 Other variables

We obtained maternal age at delivery and parity (nulliparous, multiparous) from the MBRN, and maternal educational level (≤ 12 y, 13–16 y, 17 y +), marital status (living with partner, yes/no) and number of teeth with amalgam from the self-reported baseline questionnaire answered around gestational week 17. Total energy intake was calculated from the FFQ. Information about coastal or inland residence (two categories defined by proximity of residence to the coast), health region affiliation (South-east, West, Middle, North) and year of childbirth (2002–2009) were obtained from the MoBa data managers. Since only four participants were born in 2009 these were categorized together with the birth year 2008 in the analyses.

### 2.6 Statistical methods

The analyses were performed using Stata/SE 16.0 (Statacorp, Texas).

### 2.7 Prediction model

In building the prediction model for BTHg and testing of validity of the model, only the study population with complete information was used. In the subgroup with analyzed BTHg (n = 3590) 717 participants had missing information in at least one of the covariates teeth with amalgam (n = 589), pre-pregnancy weight (n = 63), parity (n = 36), and education (n = 79), resulting in a sample of n = 2873 with complete set of variables. The sample was split into two random groups (training dataset n = 1437 and validation dataset n = 1436) using the “split sample” command in Stata.

We used linear regression to build the model of best fit for predicting BTHg concentration. The logarithm (Ln) of BTHg was the dependent variable, while the reported intake of food in groups (egg, bread and cereals, and fish and seafood in five sub-groups), smoking in pregnancy, pre-pregnancy BMI, maternal age at delivery, parity, marital status, number of teeth with amalgam, coastal or inland residence, health region affiliation, year of childbirth and total energy intake were independent variables in the model. Independent variables were selected based on previous knowledge to dietary and non-dietary sources of Hg in addition to socio-demographic and pregnancy related factors of potential importance. Variables were entered individually into the analysis and retained in the model if they contributed to increase in explained variance (R-squared) by more than 1 % and was associated with BTHg with p-value <0.05.

Two-way interactions between all variables were explored and interaction terms were included in the model if they changed the R-squared with more than 15 %. Only the interaction between maternal age and number of teeth with amalgam had an impact higher than 15 %, and the interaction term was included in the model.

### 2.8 Validation of the prediction model

The underlying linearity and constant variance assumptions in the model were checked using a diagnostic residual plot. The validity of the model was evaluated by comparing the predicted BTHg concentrations with the measured values of BTHg in in the validation dataset. The predicted log-transformed BTHg was back-transformed to original scale to evaluate the agreement between predicted and measured concentrations. We evaluated the agreement between the predicted and measured concentration by first examining the correlation using Spearman correlation coefficient. Then we used percentile plots to visually examine the mean differences in the predicted and measured concentrations, and further used a Bland-Altman plot to examine the difference between the two measurements for each subject against their mean.

The predicted and the measured blood concentrations were ranked into quintiles and the weighted Kappa (Кw) procedure was applied to evaluate the ability of the models to accurately rank the subjects.

#### 2.8.1 Prediction of BTHg in the total MoBa sample

The predicted BTHg in the women in the total MoBa sample was calculated with an out-of-sample prediction. In the subgroup with analyzed BTHg (n = 3590), the BTHg in women with missing information on number of teeth with amalgam (n = 589) did not differ from BTHg in women without missing information at similar age. Therefore, the missing values on amalgam in the MoBa study population (n = 20,152) were imputed using the mean number of teeth with amalgam in the corresponding age group. Missing information for parity (n = 1182) and education (n = 1793) in the whole MoBa population was imputed by the mean value. The prediction run in the dataset with no missing variables (n = 63,648) and predictions run in the imputed dataset (n = 86,775) showed the same explained variance. Likewise, the correlation between predicted BTHg and measured BTHg was the same using complete cases or imputed parameters.

#### 2.8.2 Analysis of the association between measured or predicted BTHg and birth weight

Multivariable linear regression was used to explore the association between maternal measured or predicted BTHg and infant birth weight. The predicted BTHg concentration (n = 86,775; imputed dataset) and the measured BTHg (n = 3590) were categorized into quintiles for the regression analyses to replicate the previous analysis of THg calculated from the maternal diet and birth weight [5].

Adjustment variables included in the analysis were those used in the previous study and excluded infants born before gestational week ≤37 and after week 42 [5]. Seafood intake was divided into the five sub-groups as described above. Maternal age at delivery, parity (nulliparous, multiparous), pre-pregnancy BMI (>18.5, 18.5–24.9, 25.0–29.9, 30.0–34.9 and 35.0 kg/m^2^), maternal educational level (≤ 12 y, 13–16 y, 17 y +), first trimester smoking (non-smoker, occasional smoker, daily smoker), total energy intake, gestational weight gain, gestational age, long-chain n-3 fatty acids intake from food and supplements were used as covariates. For calculation of long-chain n-3 fatty acids intake from food, we used data from the Norwegian food composition table, and for intake from supplements we used data from a database including more than 1000 different dietary supplements with nutrient content per portion [34].

We used *t*-test to test the difference in mean birth weight in the MoBa population and the sub-group with measured BTHg.

## 3 Results

### 3.1 Measured BTHg, dietary intake and maternal characteristics

The median and mean BTHg concentration in the study sample with measured concentrations (n = 3590) was 1.05 μg/L and 1.28 μg/L, respectively (Table 1). BTHg increased with increasing fish consumption. This applied to total seafood consumption as well as all sub-groups of seafood except salmon and trout. Furthermore, BTHg increased with increasing number of teeth with amalgam fillings. Mothers living in coastal communities had higher BTHg than mothers living in inland communities. Also, mothers living in the western and northern parts of Norway had higher mean BTHg than those living in the southeastern and middle parts. BTHg also increased with maternal age and educational attainment.

**Table 1:**
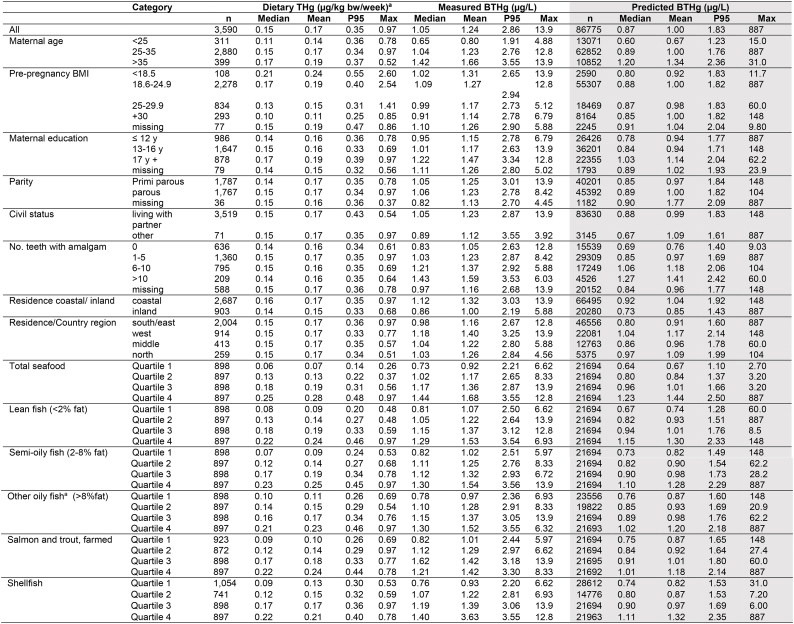
Calculated dietary intake of total mercury (Dietary THg μg/kg bw/week), concentration of total mercury in maternal blood (BTHg, μg/L) in a subgroup of MoBa participants (n = 3590) and predicted concentration of total mercury in maternal blood (BTHg μg/L) in the MoBa study population (n = 86,775), shown by maternal characteristics and reported fish and seafood consumption.

The mean and median weekly calculated dietary Hg intake in the study sample with measured BTHg was 0.17 and 0.15 μg/kg bw/week, respectively, and the highest value was 0.97 μg/kg bw/week. The mean dietary THg intake increased with increasing total seafood consumption, and this applied to all the five seafood sub-groups. Dietary THg intake also increased with maternal age, but was quite similar across categories of parity, number of teeth with amalgam and place of residence.

The mean daily consumption of foods (Table S1) and the corresponding estimated intake of THg in the women with measured BTHg levels indicated that the main contributor to THg intake was seafood, constituting 89 % of the mean THg intake. Bread and cereals contributed 4.6 % (rice being the main source) and eggs 1.9 %. Among the seafood categories, the consumption of lean fish contributed approximately 40 %, oily and semi-oily fish contributed approximately 50 %, whereas shellfish contributed approximately 9 % of the estimated intake of THg from total seafood in these pregnant women (Fig. S1).

The inclusion period in MoBa for the study population was 2002–2009, but the majority (79 %) of women with measured BTHg gave birth in 2004–2006 (Supplemental Table S2). In the same period the mean number of teeth with amalgam fillings decreased from 5.6 among women included in 2002 to 2.6 among those included in 2008. The mean number of teeth with amalgam also increased by maternal age, but the mean age at delivery was constant around 30 years in all years (data not shown). The mean measured BTHg concentration decreased by year of inclusion, whereas the dietary exposure was constant (Supplemental Table S2).

### 3.2 Associations between measured BTHg, estimated dietary THg and Hg determinants

Results in Table 2 show that the correlations between measured BTHg and estimated dietary THg and the different seafood categories were significant. The correlation between measured BTHg and estimated dietary THg was similar to the correlation between measured BTHg and seafood intake (Spearman Rho of 0.36 and 0.35, respectively). Measured BTHg also correlated significantly with the number of teeth with amalgam but there was no correlation between number of teeth with amalgam and total seafood intake (Table 2).

**Table 2:** Correlation (Spearman Rho) between measured BTHg, number of teeth with amalgam, estimated dietary THg and categories of reported seafood intake.

	BTHg (μg/L)	Teeth with amalgam	THg diet (μg/kg bw/week)^a^	Total seafood (g/day)	Lean fish (g/day)	Semi-oily fish (g/day)	Other oily fish^b^ (g/day)	Salmon and trout (g/day)	Shellfish (g/day)
BTHg (μg/L)	**1**	0.22^c^	0.34^c^	0.35^c^	0.25^c^	0.23^c^	0.24^c^	0.19^c^	0.33^c^
Teeth with amalgam	0.22^c^	**1**	−0.02	0.02	0.05	−0.03	<-0.01	−0.04	−0.06
THg diet (μg/kg bw/week)^a^	0.34^c^	−0.02	**1**	0.84^c^	0.63^c^	0.62^c^	0.45^c^	0.59^c^	0.32^c^
Total seafood (g/day)	0.35^c^	0.02	0.84^c^	**1**	0.74^c^	0.48^c^	0.61^c^	0.47^c^	0.33^c^
Lean fish (g/day)	0.25^c^	0.05	0.63^c^	0.74^c^	**1**	0.25^c^	0.22^c^	0.25^c^	0.05^d^
Semi-oily fish (g/day)	0.23^c^	−0.03	0.62^c^	0.48^c^	0.25^c^	**1**	0.18^c^	0.73^c^	0.18^c^
Oily fish^b^ (g/day)	0.24^c^	<-0.01	0.45^c^	0.61^c^	0.22^c^	0.18^c^	**1**	0.22^c^	0.18^c^
Salmon and trout (g/day)	0.19^c^	−0.04	0.59^c^	0.47^c^	0.25^c^	0.73^c^	0.22^c^	**1**	0.17^c^
Shellfish (g/day)	0.33^c^	−0.06	0.32^c^	0.33^c^	0.05^d^	0.18^c^	0.18^c^	0.17^c^	**1**

a Pre-pregnancy body weight (bw) was missing for n = 63 and has been imputed as mean pre-pregnancy bw in the sample.

b Other oily fish, incudes fish liver and roe. Bonferroni adjusted significance levels.

c P < 0.001.

d p < 0.02.

### 3.3 Prediction and validation of BTHg

The prediction model that was built based on the linear regression model from the training dataset (n = 1437 R-squared = 0.3, Supplemental Table S3) is described by the following equation (estimated β values are shown in Supplemental Table S3):

Ln BTHg = Constant + β_1_*lean fish consumption_[g/day]_ + β_2_*semi-oily fish consumption_[g/day]_ + β_3_*oily fish (not farmed) consumption_[g/day]_ + β_4_*salmon and trout consumption_[g/day]_ + β_5_*shellfish consumption_[g/day]_ + β_6_*maternal age_[years]_ + β_7_* teeth with amalgam _[no of teeth]_ + β_8_*maternal age_[years]_ * teeth with amalgam _[no of teeth]_ + β_9_*maternal education_[<12y (ref.), 13-16y, 17y+]_ + β_10_*parity_[primiparous (ref.),1, 2, 3 or more]_ + β_11_*living coast/inland_[costal (ref.), inland]_ + β_12_*health region affiliation_[North, South/East (ref.), West, Middle]_ + β_13_*total energy intake_[kcal]_ + β_14_*year of child birth_[2005 (ref.), 2002-2008]_ + β_15_*civil status _[single (ref.), living with partner]_

In the validation dataset (n = 1436), the predicted BTHg concentrations were strongly associated with the measured BTHg concentrations (Spearman correlation r = 0.53 p < 0.001, Supplemental Table S4). After imputation of missing values in the subgroup with measured BTHg, the correlation between the predicted and the measured BTHg was 0.54 (Spearman's rho, n = 3590).

**Fig. 1:**
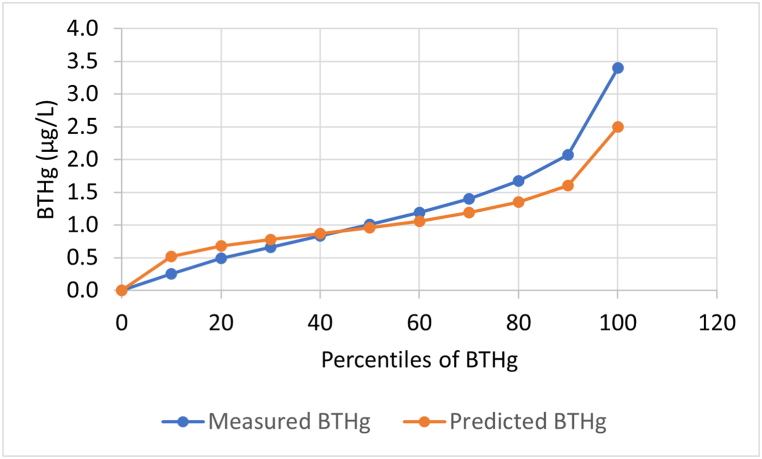
Percentile plot showing mean of predicted and measured BTHg (blood total mercury) in deciles in the validation sample (n = 1436).

The measured and predicted BTHg in deciles in the validation sample, show that the prediction model tend to overestimate the BTHg in the lower deciles and to underestimate BTHg in the higher deciles (Fig. 1 and Supplemental Table S5).

The difference between the predicted and the measured BTHg increased with increasing mean of predicted and measured BTHg, but was centered around 0, and the predicted concentration showed less precision with increasing predicted values (Supplemental Table S4).

To evaluate the ability of the model to accurately rank the subjects, the predicted and the measured BTHg concentrations were ranked into quintiles (Supplemental Table S6). Cross-classification of individuals by quintiles of measured and predicted BTHg concentrations showed that 73 % were classified into same or adjacent quintiles (correctly classified), and 3.3 % were classified into opposing quintiles (grossly misclassified). The weighted Kappa was 0.37.

### 3.4 Predicted and measured maternal BTHg and association with birth weight

The predicted BTHg in the MoBa study population (n = 86,775) showed a mean of 1.00 μg/L (range 0.10–887 μg/L) (Table 1). The correlation between the calculated dietary THg (μg/day) and the predicted BTHg was 0.54 (Spearman's rho, n = 86,775).

In total, 18 of the participants were predicted to have BTHg above the highest measured BTHg (13.9 μg/L). The predicted BTHg in these ranged from 14.3 to 887 μg/L, and three of the predicted values exceeded 100 μg/L. These participants were all in the highest quantile of reported seafood intake and calculated dietary THg exposure. Most of these participants reported a high shellfish intake and lived in coastal areas (16 of 18).

In pregnant women with measured BTHg (n = 3588) and predicted BTHg (n = 86775) we investigated the associations between BTHg exposure categorized into quintiles and child birth weight (Table 3). In the group with measured BTHg, we did not identify any associations between measured BTHg and birth weight. In the group with predicted BTHg, BTHg was statistically significant associated with higher birth weight in quintile 3, 4 and 5, and most prominently in quintile 5.

**Table 3:** Associations between measured or predicted blood total Hg concentrations (BTHg) in pregnant women and birth weight in gram (g) in the MoBa study.

	Quintiles (range) μg/L	n	Unadjusted		Adjusted^a^	
			β (g)	95 % CI	β (g)	95 % CI
**Measured** BTHg n = 3588	Q 1 (<0.56)	721	referent	–	referent	–
	Q 2 (0.56–0.90)	718	10	(-42,62)	14	(-30,59)
	Q 3 (0.90–1.3)	715	−40	(-95,14)	−32	(-78,13)
	Q 4 (1.3–1.8)	717	14	(-39,67)	34	(-12,81)
	Q 5 (1.8–13.7)	717	10	(-42,63)	31	(-19,81)
**Predicted** BTHg n = 86,775	Q 1 (<0.61)	17,355	referent	–	referent	–
	Q 2 (0.61–0.78)	17,355	22	(10,35)	9	(-0.8,19)
	Q 3 (0.78–0.97)	17,355	35	(23,48)	32	(22,43)
	Q 4 (0.97–1.26)	17,355	22	(9,34)	30	(18,41)
	Q 5 (1.26–887)	17,355	31	(19,44)	62	(48,77)

a Adjusted for seafood intake in sub-groups, energy intake, maternal age, pre-pregnancy BMI, parity, smoking during pregnancy, intake of n-3 fatty acids, maternal education, gestational age, gestational weight gain.

**Fig. 2:**
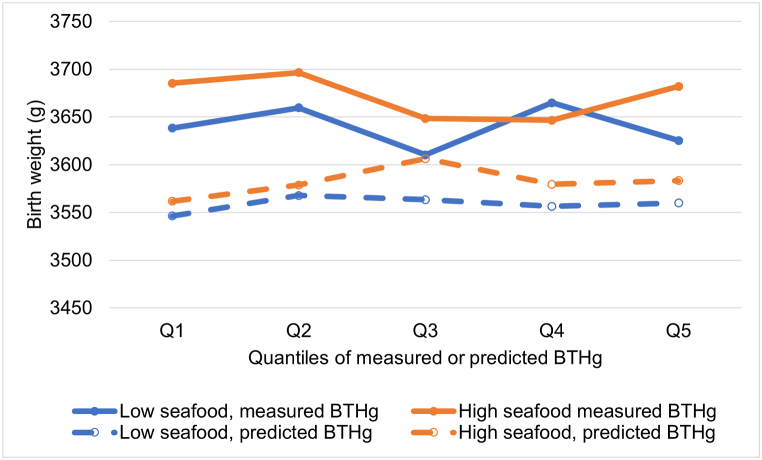
Mean birth weight in quintiles of measured BTHg (n = 3588) or predicted BTHg (n = 83,086) in low (below the median of 31.2 g per day) or high (above median) reported seafood consumption during pregnancy in MoBa. BTHg: Blood total mercury.

Participants were divided into high or low seafood consumers (reporting total seafood consumption above or below the median of 31.2 g per day in the MoBa population) and mean birth weight in quintiles of measured and predicted BTHg was plotted (Fig. 2). There were no observed trends in birth weight with increasing quintiles of measured or predicted BTHg. In general, the women with fish consumption above the median during pregnancy gave birth to children with higher mean birth weight than the women with fish consumption below the median. Notably, the mean birth weight in the women with measured BTHg (n = 3588) was 88 g higher than in the women with predicted BTHg (n = 83086, p < 0.001). Mean (SD) birth weight, and n in each quintile is shown in Supplemental Table S7.

## 4 Discussion

The model developed for predicting BTHg in the present study showed that four maternal seafood intake sub-groups (lean fish, semi-oily fish and oily fish except salmon and trout, shellfish), number of teeth with amalgam, age, coastal or inland residence, year of childbirth, education level, parity, marital status and energy intake are important determinants of BTHg in pregnant women in MoBa. The validation of the model, comparing predicted and measured BTHg concentrations in pregnant MoBa women in the validation sample, showed moderate accuracy, with a tendency for overestimation in low percentiles and underestimation in high percentiles of measured BTHg. We further used the model to predict BTHg in the full MoBa study sample. In contrast to what we expected based on previous results, which indicated inverse relationship between dietary Hg intake and birth weight in strata of fish consumption [5], the predicted BTHg was associated with increased birth weight. Furthermore, we found no association between measured BTHg and birth weight. When the participants were stratified into low and high reported seafood consumption, there was no difference in birth weight across quintiles of predicted and measured BTHg.

### 4.1 Determinants of BTHg

Fish consumption is a well-known determinant of BTHg, and all the seafood categories except farmed salmon and trout were significant predictors in the present study. Although the mean consumption of farmed salmon and trout (2.8 g per day) was quite similar to semi-oily fish (3.1 g per day), salmon and trout contributed only 9 % whereas semi-oily fish contributed 22 % to the THg intake, in the study population with measured BTHg (Supplemental Table S1 and Supplemental Fig. S3). Also, the estimated dietary intake of THg showed a similar correlation with measured BTHg as the fish categories. The validation of the MoBa FFQ included measurement of BTHg in a group of 119 pregnant women in MoBa [33]. The correlation between estimated total fish and seafood intake and measured BTHg in these 119 women was moderate (0.36, 95 % CI: 0.19, 0.51) [31]. When the same FFQ was used in another study-population that included men and women aged 21–80 years who had a much wider range of fish consumption, the correlation between total seafood consumption and measured BTHg was 0.58 (95 % CI: 0.48, 0.67) [12]. The median seafood consumption (65 g/day) was more than two times higher than in the present study (31 g/day).

Dental amalgam is a known contributor to measured BTHg concentration, providing IHg [35]. Of note, the inclusion period in MoBa was 2002–2009. In the same time period the mean number of teeth with amalgam fillings decreased among the participants (Supplemental Table S2), coinciding with the phasing out of amalgam use in Norway, which was banned in 2008 [36]. Mercury emissions decreased in Norway in the inclusion period and this may explain why year of child birth was significant in our prediction model. The interaction between the number of teeth with amalgam and the maternal age at delivery may be explained by an accumulation of number of teeth with amalgam by age. We have not identified data that indicate a time trend in Hg in the fish species mostly consumed in Norway apart from a small decrease in farmed salmon, likely due to a reduction in the inclusion of fish meal in the fish diet [[36], [37], [38]].

Coastal residence has previously been shown to be associated with higher Hg exposure [12]. A higher seafood consumption in coastal aeras should be captured by the FFQ, but in addition, access to local seafood caught near land may contain higher mercury concentrations than seafood originating from the open sea [12,39]. The health region the participating women belong to was significant in our model, and we believe that this variable reflects the access to local seafood in combination with concentration of THg in local seafood, which is not captured in databases on concentrations of THg in seafood.

### 4.2 BTHg in Norway and in other countries

Despite a relatively high seafood consumption among the MoBa women in this study (mean 33 g/day Supplemental Table S1), compared to adults in most surveys in Europe available to EFSA (EFSA comprehensive food consumption database [40]) the median measured BTHg in our participants (1,05 μg/L) is below that in women in other European countries with ample access to seafood, showing median or geometric mean BTHg of 1.1–6.3 μg/L [[41], [42], [43], [44]]. The reason that the measured BTHg is low despite relatively high fish consumption is most likely that the fish species commonly eaten in Norway (cod, farmed salmon and mackerel) contain low levels of Hg [12]. The highest measured BTHg concentration in our sample (13.6 μg/L) is considerably lower than 23 μg/L, which is the maternal blood concentration calculated by EFSA to correspond to the no observed adverse effect level in maternal hair (THg 11.5 mg/kg hair) when including an extra factor of 2 to account for the uncertainty in the hair to blood ratio of 250, and which was used as basis for setting the TWI for MeHg in 2012 [2].

### 4.3 Validation of the prediction model

The measured and predicted BTHg levels in deciles in the validation sample indicated that the model tends to overestimate BTHg in the lower deciles and to underestimate BTHg in the higher deciles. One possible explanation for this may be that some high fish consumers obtain their seafood from local suppliers or are catching fish themselves close to the shore, and this fish is possibly higher in MeHg than fish obtained from the open sea, which is available in stores. We have previously observed that self-catching is associated with higher BTHg [12]. Such deviations in predicted from observed BTHg may lead to less difference between categories of low and high predicted BTHg in the larger MoBa sample, possibly hampering identifications of associations with health outcomes. However, the validation of the prediction model with a weighted Kappa of 0.37 indicated that the model can predict BTHg in MoBa with a moderate accuracy [45]. The predicted BTHg was able to rank participants into high and low exposure, and <5 % grossly misclassified, which was the case in this study, is considered good (Supplemental Table S6). Thus, we found it reasonable to use predicted BTHg in a larger MoBa sample when investigating associations between BTHg and birth weight.

### 4.4 Association between BTHg and birth weight

In our previous study showing an inverse association between dietary Hg and birth weight, there was a decreasing trend in birth weight across quantiles of fish intake [5], and similar associations were explored in the present study (Fig. 2).

The predicted mean BTHg in the MoBa study population (1.00 μg/L, n = 86,775) was lower than the mean BTHg in the women with measured BTHg (1.24 μg/L, n = 3590) as shown in Table 1. Having complete data for questionnaires was an inclusion criterion for being selected for BTHg analysis in the MoBa E-tox sample, which constitute the majority in our study group with analyzed BTHg, and women in this group tended to have longer education than the total MoBa sample [25]. Since fish consumption is positively associated with higher education in MoBa this might explain the lower predicted BTHg levels in the larger MoBa sample. Furthermore, and in line with this, the mean birth weight in the larger MoBa sample with predicted BTHg was lower than in the group of women with measured BTHg (Fig. 2).

Available literature data on associations between measured maternal or foetal BTHg concentration and birth weight were not conclusive in the risk assessment of MeHg conducted by EFSA [2]. A more recent narrative review based on a systematic literature search concluded there was some evidence of negative association of measured biomarkers of mercury exposure and birth weight [3]. Another recent narrative review based on a systematic literature search also came to a similar conclusion, but the authors also noted that Hg toxicity may sometimes be mitigated by e.g. polyunsaturated fatty acids in the maternal diet [4].

We have previously reported that total fish consumption is associated with higher birth weight in MoBa, but also reported that increasing Hg intake across different strata of seafood consumption was associated with a decrease in birth weight ([5,46]). We explored whether the predicted BTHg could provide sufficiently-sized study sample in MoBa to reproduce the observed negative association between dietary intake of THg and birth weight at low exposure, given that it provided estimates of BTHg levels, which could be statistically adjusted for factors affecting birth weight. The analyzed BTHg in the subsample was not associated with birth weight, and the association with predicted BTHg indicated higher birth weight by higher BTHg, although not clearly dose related. These findings are in line with our previous results that fish consumption is associated with higher birth weight in MoBa [46]. Of note, positive (apparently “beneficial”) associations of BTHg and neurodevelopmental outcomes after adjustment for fish consumption have previously been observed in MoBa [17,18,47], and it can be speculated that measured BTHg provides a better marker of seafood consumption than self-reported seafood consumption from the FFQ in pregnant MoBa women, thus limiting the ability of this seafood variable to fully adjust for the negative confounding.

Our results do not indicate that measured or predicted BTHg concentrations are associated with lower birth weight in our study population. Rather contrary, higher predicted BTHg is associated with higher birth weight, and furthermore, higher fish consumption is also associated with higher birth weight, as illustrated in Fig. 2. This may indicate that the application of predicted BTHg in regression models adjusted for fish consumption does not overcome the obstacles of negative confounding. This might be because the major determinants in the model developed for predicting BTHg in MoBa included the same food items that contribute most to estimated dietary THg intake. Consequently, a possible positive association between fish consumption and birth weight could not be separated from a potential negative association between MeHg in seafood and birth weight, preventing us from concluding that fish consumption would have been more beneficial if the MeHg concentration would have been lower. However, since there was no indication of any association between BTHg and lower birth weight in the relatively large subsample of n = 3588 with measured BTHg, any potential negative association in the observed range of BTHg in MoBa is probably weak (small effect sizes). So far, efforts to disentangle toxic effects of Hg and beneficial effects of seafood has proven to be challenging in relation to e.g. cognitive development at much higher Hg exposure levels than in the present study [48,49].

### 4.5 Strengths and weaknesses

Our study has several strengths and weaknesses. The relatively large subgroup with measured BTHg concentrations enabled dividing into a prediction sample and a validation sample. Furthermore, the large number of participants in the full MoBa cohort represent women from all parts of Norway. We see it as a strength that few of the women in the larger MoBa sample had predicted BTHg above the highest measured BTHg in the subgroup with measured BTHg, so that few have predicted BTHg outside observed range. Another strength in our study is the detailed and validated FFQ which was specifically designed to capture foods with a high content of environmental contaminants. However, FFQs are rather crude instruments which are prone to misreporting and recall bias. The low participation rate in MoBa is a concern as participants are older, have higher education and include fewer smokers than the general population of pregnant women [50]. Furthermore, women in the subgroup with measured BTHg are even less representative of the general population of pregnant women. The lack of measured urinary Hg, being a marker of IHg exposure can be seen as a weakness. On the other hand, studies have shown that the number of teeth with amalgam contributes substantially to the total IHg exposure, and urinary Hg also reflects fish intake, due to demethylation of MeHg [44,51]. Furthermore, we lack information on any work-related exposure to Hg. Finally, the ranges of both the predicted and measured BTHg are rather narrow in this group of pregnant women, which reduce the ability to detect weak associations.

## 5 Conclusion

This study showed that the major determinants in the model developed for predicting BTHg in MoBa included the same food items that contribute most to estimated dietary THg intake. The agreement between the predicted and measured BTHg was moderate. We hypothesized that predicted BTHg would be associated with decreased birth weight based on earlier findings. However, we observed that predicted BTHg was associated with increased birth weight, which is in line with a previous study based on maternal seafood intake in MoBa. This result could indicate that negative confounding of the association between Hg exposure and birth weight by seafood intake was not overcome by prediction of BTHg. Furthermore, the lack of association between measured BTHg and birth weight in the sub-sample of 3590 pregnant women may indicate that if this range of relatively low BTHg is associated with lower birth weight, the association is weak with small effect size and is likely not of toxicological relevance.

## Funding

The Norwegian Mother, Father and Child Cohort Study is supported by the Norwegian 10.13039/501100003506Ministry of Health and Care Services and the Ministry of Education and Research. The Norwegian Institute of Public Health (NIPH) has contributed to funding of the Norwegian Environmental Biobank. IHC received funding under The Norwegian Research Council's Centers of Excellence Funding Scheme, grant no. 262700.

## Data availability statement

Data associated with this study has not been deposited into any publicly available repository because the authors do not have permission to share the data.

## CRediT authorship contribution statement

**Kristine Vejrup:** Writing – review & editing, Visualization, Validation, Methodology, Formal analysis, Conceptualization. **Anne Lise Brantsæter:** Writing – review & editing, Validation, Project administration, Methodology, Formal analysis, Conceptualization. **Ida H. Caspersen:** Writing – review & editing, Validation, Methodology, Formal analysis, Conceptualization. **Line S. Haug:** Writing – review & editing, Resources. **Gro D. Villanger:** Writing – review & editing, Resources. **Heidi Aase:** Writing – review & editing, Resources. **Helle K. Knutsen:** Writing – review & editing, Writing – original draft, Visualization, Validation, Methodology, Conceptualization.

## Declaration of competing interest

All authors declare that they have no known competing financial interests or personal relationships that could have appeared to influence the work reported in this paper.
