# Supplementary material for: Mercury exposure in the Norwegian Mother, Father, and Child Cohort Study – measured and predicted blood concentrations and associations with birth weight
Source: Heliyon. 2024 Apr 25;10(9):e30246. doi: 10.1016/j.heliyon.2024.e30246 (PMC11078626; doi:10.1016/j.heliyon.2024.e30246)
Supplement: Multimedia component 1 [file mmc1.docx]

# Mercury exposure in the Norwegian Mother, Father, and Child Cohort Study – measured and predicted blood concentrations and associations with birth weight.

# Supplementary material

**Table S1.** Estimated daily consumption of food in food groups (g/day) and calculated mean dietary THg intake per food group (µg/day) in 3,590 pregnant women participating in the Norwegian Mother, Father and Child Cohort Study.

|  | **Food consumed (g/day)** | | **Calculated THg (µg/day)** | |
| --- | --- | --- | --- | --- |
| **Food groups** | **mean** | **min-max** | **mean** | **min-max** |
| **Total diet** | n.a. | n.a. | 1.57 | 0-8.25 |
| **Total Seafood** | 33.5 | 0-208 | 1.40 | 0-8.11 |
| *-Lean fish* | *16* | *0-88* | *0.56* | *0-6.4* |
| *-Semi oily fish* | *3.1* | *0-40* | *0.30* | *0-2.8* |
| *-Salmon and trout* | *2.8* | *0-37* | *0.13* | *0-1.2* |
| *-Other oily fish* | *7.7* | *0-163* | *0.28* | *0-3.8* |
| *-Shellfish* | *4.1* | *0-94* | *0.13* | *0-3.9* |
| **Bread and cereals** | 316 | 0-859 | 0.07 | 0-0.55 |
| **Eggs** | 17.1 | 0-147 | 0.03 | 0-0.41 |
| **Other foods** | 3186 | 0-13813 | 0.02 | 0-0.56 |

Lean fish (<2% fat, e.g. cod, saithe), semi-oily fish (2-8% fat, e.g. wolffish, halibut, flounder), salmon and trout (predominantly farmed), other oily fish than salmon/trout (>8% fat, e.g. mackerel, herring, also including liver and roe), shellfish (scrimps, crabs and bivalves). n.a.: not applicable.

**Table S2.** Mean concentration of measured BTHg, dietary intake of Hg and number of teeth with amalgam by childbirth year in the sample of 3,590 MoBa women.

| **Birth year** | **N** | **%** | **BTHg mean (µg/L)** | **Dietary THg intake (µg/kg bw per week)** | **Dietary THg intake from fish**  **(µg/kg bw per week)** | **Number of teeth** with amalgam** |
| --- | --- | --- | --- | --- | --- | --- |
| 2002 | 183 | 5.1 | 1.320 | 0.17 | 0.15 | 5.6 |
| 2003 | 239 | 6.7 | 1.266 | 0.16 | 0.14 | 5.1 |
| 2004 | 890 | 24.8 | 1.298 | 0.16 | 0.14 | 4.9 |
| 2005 | 1327 | 37.0 | 1.288 | 0.17 | 0.15 | 4.2 |
| 2006 | 613 | 17.1 | 1.119 | 0.17 | 0.15 | 3.7 |
| 2007 | 201 | 5.6 | 1.042 | 0.17 | 0.15 | 3.3 |
| 2008* | 137 | 3.8 | 0.985 | 0.19 | 0.17 | 2.6 |

*Including n=4 in 2009 **Missing information: n=589 women

**Table S3.** Linear regression model built on the training dataset (n=1,437).

| R squared 0.30, adjusted R squared 0.29 | | | |
| --- | --- | --- | --- |
|  | Coefficient | 95% CI | P |
| Constant | -2.221 | -2.663, -1.779 | <0.001 |
| Lean fish (g per day) | 0.0143 | 0.0110, 0.0176 | <0.001 |
| Semi-oily fish (g per day) | 0.0198 | 0.00861, 0.0310 | 0.001 |
| Oily fish, other than salmon/trout (g per day) | 0.00466 | 0.00194, 0.00738 | 0.001 |
| Salmon and trout (mostly farmed) (g per day) | 0.00277 | -0.00925, 0.0148 | 0.45 |
| Shellfish (g per week) | 0.0306 | 0.0239, 0.0373 | <0.001 |
| Maternal age at delivery | 0.0505 | 0.0385, 0.0626 | <0.001 |
| Number of teeth with amalgam | 0.110 | 0.0550, 0.166 | <0.001 |
| Number of teeth with amalgam * maternal age at delivery | -0.00262 | -0.00436, -0.000895 | 0.003 |
| Maternal education |  |  |  |
| <12 years | Reference |  |  |
| 13-16 years | 0.0191 | -0.0603, 0.0984 | 0.47 |
| >17 years | 0.195 | 0.102, 0.289 | <0.001 |
| Parity |  |  |  |
| Primiparous | Reference |  |  |
| 1 | -0.141 | -0.217, -0.0650 | <0.001 |
| 2 | -0.155 | -0.266, 0.0444 | 0.006 |
| 3 or more | -0.270 | -0.478, -0.0625 | <0.001 |
| Inland vs coastal residence |  |  |  |
| Coastal | Reference |  |  |
| Inland | -0.120 | -0.201, -0.0385 | 0.004 |
| Regional health affiliation |  |  |  |
| East | Reference |  |  |
| West | 0.178 | 0.0952, 0.261 | <0.001 |
| Mid | 0.0689 | -0.0395, 0.177 | 0.21 |
| North | 0.0217 | -0.117, 0.161 | 0.31 |
| Total energy intake (kcal) | -0.0000947 | -0.000146, -0.0000431 | <0.001 |
| Year of childbirth |  |  |  |
| 2002 | 0.148 | -0.00492, 0.296 | 0.05 |
| 2003 | 0.050 | -0.0863, 0.186 | 0.47 |
| 2004 | 0.0644 | -0.194, 0.148 | 0.13 |
| 2005 | Reference |  |  |
| 2006 | -0.163 | -0.256, -0.0715 | 0.001 |
| 2007 | -0.237 | -0.387, -0.873 | 0.001 |
| 2008 | -0.282 | -0.463, -0.0998 | 0.002 |
| Marital status |  |  |  |
| Single | Reference |  |  |
| Living with partner | 0.361 | 0.117, 0.606 | 0.004 |

**Table S4.** Measured and predicted BTHg in the validation sample (n=1,436). Spearman’s correlation coefficient with Bonferroni adjusted significant levels.

|  | **mean** | **median** | **(p25, p75)** | **Correlation r, significance** |
| --- | --- | --- | --- | --- |
| **Measured BTHg** | 1.29 | 1.09 | (0.65, 1.66) | 0.53, p<0.001 |
| **Predicted BTHg** | 1.15 | 1.00 | (0.79, 1.36) |  |

**Table S5.** Predicted and measured BTHg in quintiles in the validation dataset.

| **BTHg predicted** | **N** | **mean** | **min** | **max** | **sd** | **P50** |
| --- | --- | --- | --- | --- | --- | --- |
| 1 quintile | 288 | 0.60 | 0.30 | 0.75 | 0.11 | 0.62 |
| 2 quintile | 287 | 0.83 | 0.75 | 0.92 | 0.05 | 0.83 |
| 3 quintile | 287 | 1.02 | 0.92 | 1.12 | 0.06 | 1.01 |
| 4 quintile | 287 | 1.28 | 1.13 | 1.45 | 0.10 | 1.27 |
| 5 quintile | 287 | 2.04 | 1.45 | 7.00 | 0.70 | 1.81 |
| total | 1436 | 1.15 | 0.30 | 7.00 | 0.59 | 1.01 |
| **BTHg measured** | **N** | **mean** | **min** | **max** | **sd** | **P50** |
| 1 quintile | 288 | 0.37 | 0.003 | 0.57 | 0.14 | 0.38 |
| 2 quintile | 288 | 0.74 | 0.57 | 0.91 | 0.10 | 0.75 |
| 3 quintile | 286 | 1.09 | 0.91 | 1.27 | 0.10 | 1.09 |
| 4 quintile | 288 | 1.52 | 1.28 | 1.82 | 0.16 | 1.51 |
| 5 quintile | 286 | 2.71 | 1.82 | 12.68 | 1.10 | 2.35 |
| total | 1436 | 1.29 | 0.003 | 12.68 | 0.95 | 1.09 |

**Table S6.** Cross classification of individuals, n (%), by quintiles of measured and predicted blood total Hg concentrations (BTHg) in the validation sample.

|  | **Predicted BTHg** | | | | | |
| --- | --- | --- | --- | --- | --- | --- |
| **Measured BTHg** | **Q1** | **Q2** | **Q3** | **Q4** | **Q5** | **Total n** |
| **Q1** | 160 (55.6%) | 56 (19.4%) | 39 (13.6%) | 21 (7.3%) | 12 (4.2%) | 288 |
| **Q2** | 63 (21.9%) | 89 (30.9%) | 58 (20.3%) | 51 (17.7%) | 26 (9.1%) | 287 |
| **Q3** | 41 (14.2%) | 67 (23.3%) | 54 (18.9%) | 73 (25.3%) | 52 (18.2%) | 287 |
| **Q4** | 17 (5.9%) | 51 (17.7%) | 82 (28.7%) | 78 (27.1%) | 59 (20.6%) | 287 |
| **Q5** | 7 (2.4%) | 25 (8.7%) | 53 (18.5%) | 65 (22.6%) | 137 (47.9%) | 287 |
| **Total n** | 288 | 288 | 286 | 288 | 286 | 1436 |

**Table S7:** Mean birth weight (BW) in quintiles and n with measured and predicted BTHg in each quintile, stratified into low or high by the median (31.2 g/day) reported maternal seafood consumption.

|  | Measured blood Hg | | | Predicted blood Hg | | |
| --- | --- | --- | --- | --- | --- | --- |
|  | n | Mean birthweight (g) | SD | n | Mean birthweight (g) | SD |
| Low seafood consumption (< 31.2 g per day) | | | | | | |
| BW Q1 | 530 | 3645 | 539.4 | 14191 | 3546 | 577.1 |
| BW Q2 | 409 | 3662 | 482.8 | 11474 | 3568 | 571.4 |
| BW Q3 | 377 | 3585 | 522.4 | 9098 | 3564 | 607.8 |
| BW Q4 | 300 | 3694 | 524.2 | 6310 | 3556 | 606.6 |
| BW Q5 | 222 | 3635 | 509.7 | 2261 | 3560 | 619.9 |
| High seafood consumption (≥ 31.2 g per day) | | | | | | |
| BW Q1 | 191 | 3691 | 491.6 | 3148 | 3562 | 555.2 |
| BW Q2 | 307 | 3674 | 492.2 | 5858 | 3579 | 557.0 |
| BW Q3 | 341 | 3651 | 538.5 | 8239 | 3607 | 567.4 |
| BW Q4 | 418 | 3655 | 509.8 | 11002 | 3580 | 588.7 |
| BW Q5 | 493 | 3682 | 483.6 | 15072 | 3584 | 613.0 |


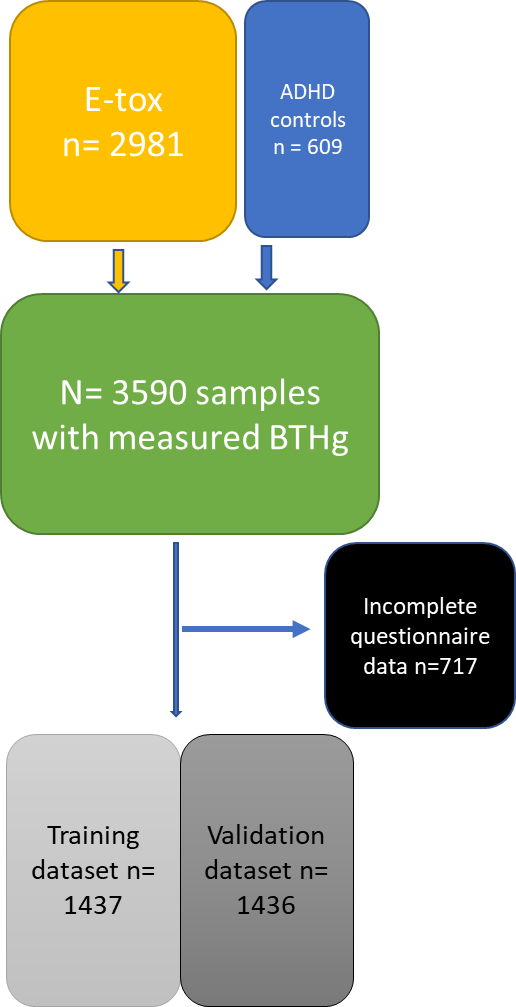


**Figure S1.** Samples for building and validating the prediction model.


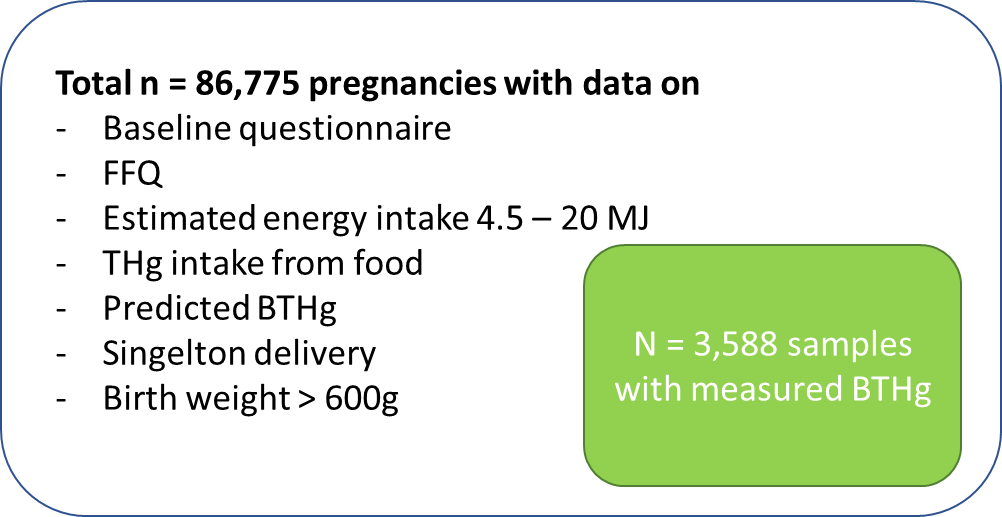


**Figure S2.** Inclusion criteria for participants in the full MoBa study sample (n=86,775) and for the sample with additional measured Hg concentration in blood (n=3,588)

**Figure S3.** Mean contribution from seafood categories to the calculated mercury intake from seafood in 3,590 pregnant women in MoBa. Lean fish (<2% fat, e.g. cod, saithe), semi-oily fish (2-8% fat, e.g. wolffish, halibut, flounder), salmon and trout (predominantly farmed), other oily fish (>8% fat, e.g. mackerel, herring, also including liver and roe), shellfish (scrimps, crabs and bivalves).

**Figure S4.** Bland Altman plot showing mean of predicted and measured concentrations of BTHg against the difference between predicted and measured BTHg in the validation sample. The grey area indicates the 95% limits of agreement (-1.841, 2.065).
